# Longitudinal effects of bilingualism on dual-tasking

**DOI:** 10.1371/journal.pone.0189299

**Published:** 2017-12-27

**Authors:** Daniel Eriksson Sörman, Maria Josefsson, John E. Marsh, Patrik Hansson, Jessica K. Ljungberg

**Affiliations:** 1 Department of Psychology, Umeå University, Umeå, Sweden; 2 Centre for Demographic and Ageing Research, Umeå University, Umeå, Sweden; 3 School of Psychology, University of Central Lancashire, Preston, United Kingdom; Utrecht University, NETHERLANDS

## Abstract

An ongoing debate surrounds whether bilinguals outperform monolinguals in tests of executive processing. The aim of this study was to investigate if there are long-term (10 year) bilingual advantages in executive processing, as indexed by dual-task performance, in a sample that were 40–65 years at baseline. The bilingual (*n* = 24) and monolingual (*n* = 24) participants were matched on age, sex, education, fluid intelligence, and study sample. Participants performed free-recall for a 12-item list in three dual-task settings wherein they sorted cards either during encoding, retrieval, or during both encoding and retrieval of the word-list. Free recall without card sorting was used as a reference to compute dual-task costs. The results showed that bilinguals significantly outperformed monolinguals when they performed card-sorting during both encoding and retrieval of the word-list, the condition that presumably placed the highest demands on executive functioning. However, dual-task costs increased over time for bilinguals relative to monolinguals, a finding that is possibly influenced by retirement age and limited use of second language in the bilingual group.

## Introduction

Across the world, the number of bilinguals is continuously increasing. Today, about half of the world’s population is considered to be either bilingual or multilingual [1]. This increase in the number of multilinguals may, from a global cognitive perspective, have positive implications. For example, a number of studies have proposed that bilingualism can postpone the onset of cognitive impairment and dementia (e.g., [2–4]). Since the average life expectancy is rapidly increasing over the world, along with the number of dementia cases, identifying the cognitive abilities that are sensitive to aging is of vital concern. Although results regarding the relationship between bilingualism and dementia are not always univocal (e.g., [5–7]), one factor that potentially obscures the relationship between bilingualism and dementia is the lower frequency of second language use in some populations when reaching an older age [8].

Results obtained from experimental settings have also shown bilingual advantages in several cognitive tasks, mainly when measuring executive control and working memory (see [9–11]). So far, most of the positive effects for bilinguals have been reported from tasks measuring executive functions such as the process of inhibition, when performing the Stroop task (e.g., [12–13]), the Flanker task (e.g., [14–15]) and the Simon Task [16]. However, beneficial effects are also reported from tasks measuring the process of switching, for example in color-shape tasks (e.g., [17–18]). It should be noted, though, that there are also studies showing a lack of relationship between bilingualism and executive processing (for recent reviews and meta-analysis see e.g., [19–22]).

Bilingual advantages seem to be particularly evident for tasks that contain non-verbal material (e.g. [12]), and benefits are less common using verbal material. This is assumed to be a result of smaller vocabulary size's in each language among bilinguals (e.g., [23–24]). One explanation for the relative lack of a bilingual advantage for tasks that incorporate verbal material is that a smaller lexical network in each language, combined with the competition between the two, causes higher load on executive functions for bilinguals when handling linguistic material. Such cognitive demands may, however, improve executive functioning and generate transfer to several cognitive tasks that do not incorporate verbal material. However, if bilinguals have relatively equal levels of ability as monolinguals in their dominant language, it is plausible that bilinguals may not be limited by tasks that include verbal material. Kaushanskaya, Blumenfeld and Marian [25], for instance, compared English–Spanish bilinguals had English as dominant language with English-speaking monolinguals using the Peabody Picture Vocabulary Test-III. Groups were matched with regard to age and education. The results showed that bilinguals not only had similar level of dominant-language vocabulary compared to monolinguals, but also comparable levels of performance in phonological short-term memory. Thus, in verbal tasks that require little attentional control, bilinguals and monolinguals may perform at the same level if they have similar skills in their dominant language.

It should be noted that positive effects of the ability to master two languages do not seem to be isolated to working memory and executive functions. For example, there is support for bilingual advantages from tasks that require episodic recall and verbal fluency skills (e.g. [26]). Beneficial effects of bilingualism, as suggested in above-mentioned studies, are of great interest considering that episodic memory (e.g., [27]), working memory (e.g., [28–29]) and executive functioning (e.g., [30]), including switching (e.g., [31–32]), inhibition (e.g., [33]) and the ability to manage interference (e.g., [34]) often decline with increasing age.

An assumption often made about bilingualism is that the executive function system has an important role in controlling which language is to be processed in different contexts [35]. Green [36] argues that bilinguals are constantly trained to inhibit the language that they are not presently using. This language suppression is assumed to co-opt the same executive functions as those used in controlled attention, and thus, it is believed that the “cognitive exercise” bilingualism engenders can generate transfer to other cognitive tasks. In addition, constant control of two language networks requires not only proficient inhibitory control, but also the ability to switch between and monitor information from different language systems [37]. Thus, it is possible that the broad training of executive functions that bilingualism involves can be extended to several tasks that require executive control, and hence also found in tasks that require dual-tasking, since the executive system is central to attention-related tasks [38].

Results from imaging studies provide a possible neuroscientific explanation for the bilingual advantage. Abutalebi et al [39] found, for instance, that the anterior cingulate cortex (ACC) was active during both cognitive control and language control among bilinguals, and more efficiently used compared to monolinguals, as they performed a number of non-verbal tasks. In addition, the ACC basal ganglia networks have been found to be important for parallel conflict monitoring and flexible adaptation of actions [40] and it is well established that the basal ganglia plays a critical role in language control processes among bilinguals when they use one language and inhibit interference from the unintended language [41]. Furthermore, based on data from other neuroimaging studies, language processing seems to be supported by frontal brain regions equivalent to those active when performing other executive tasks (see [39, 42]). Thus, it is plausible that the use of different languages in everyday life activates and improves the efficiency of brain regions used also for tasks that require controlled attention.

One area that has received scant scrutiny is how well a frequently practiced switching ability between two languages can be transferred to, and found in, dual-tasking situations. Factors promoting dual-tasking are of significant importance from an aging perspective, since the ability to dual-task has been shown to be sensitive to aging (for review see: [43]). Dual-tasking can be defined as two tasks that are performed simultaneously, that are independent of one another, that can be measured separately, and which have separate goals [44]. Efficient dual-tasking is dependent on executive functioning. Executive functioning allows the individual to follow given rules, allocate attention to the two tasks to be carried out, and to efficiently shift attentional resources as the tasks continues [45]. Dual-tasking can thus be regarded from a switching perspective, taking into account the time spent on one task before switching to the other. From this point of view, tasks that require almost simultaneous attention (i.e. dual-tasking) can be considered as a form of rapid switching [46]. In addition, Salthouse and Miles [32] suggest that the ability to solve dual-task challenges depends to a great extent on the ability for monitoring. The possible link between dual-tasking and bilingualism becomes particularly interesting since both switching and monitoring are also important aspects in managing different lexical networks [37].

In a previous study, Fernandes, Craik, Bialystok, and Kreuger [47] used a free recall task to examine if bilinguals were better at dealing with divided attention during encoding and retrieval of words. In the primary free recall task, to-be remembered words were presented auditorily and in a visual distraction task participants made size decisions to words presented on a screen. The words presented were either semantically related (e.g. remember animal words / make size decisions of animals) or unrelated (e.g. animals / fruits) in the dual-task conditions. The authors found that both older (*n* = 26, *M* = 70.1 years) and younger (*n* = 26, *M* = 20.5 years) bilinguals not only performed worse during full attention (no distraction task), but also while distracted during both encoding and retrieval (in both related and unrelated word conditions). The authors speculated that this difference may due to a bilingual disadvantage in tasks that require lexical access in one language, since they have two language networks to control.

A similar study conducted by the same research group [37] used classification tasks to examine potential differences in dual-tasking between monolinguals and bilinguals. The study aimed to examine the assumption that bilinguals have a better capacity for monitoring and switching and that these factors are typically accessed when included in dual-tasking situations. In one classification task, participants were told to categorize letters and numbers, or animals and instruments, which were visually presented on a screen. One computer-driven mouse was placed on each side of the display, and participants categorized objects by pressing on the mouse that represented each category. In the other classification task, participants heard words or sounds and verbally categorized these as “letter”/”number” or “animal”/“music”. The items were either from the same category as the one used in the visual task (related) or items from the other category (unrelated). The experiments included both younger (*n* = 24, *M* = 21.7 years for bilinguals; *n* = 24, *M* = 21.2 years for moderate bilinguals; *n* = 24, *M* = 22.7 for monolinguals), and older participants (*n* = 24, *M* = 63.2 years for bilinguals; *n* = 24, *M* = 64.7 for monolinguals). The results from this study demonstrated a bilingual advantage: Both younger and older bilinguals generally performed better than the monolinguals and this was more evident in the conditions wherein the primary task was to visually categorize letters and numbers.

The bilingual advantage in dual-tasking has on at least one occasion also been found in a context with higher ecological validity than the hitherto mentioned studies. For example, Telner, Wiesenthal, Bialystok, and York [48] showed that bilingual (*n* = 47) university students, 18–30 years, performed significantly better than monolinguals (*n* = 35) in attentional tasks including simulated car driving while performing various verbal tasks such as talking on a cell phone. Based on the findings from this study and those by Fernandes et al. [47] and Bialystok et al. [37] it could be concluded that the nature of the task as well as the choice of modality in which the to-be remembered material is presented is of high importance for the outcome.

Although there is some support for the notion that bilinguals are better at dual-tasking, in the light of aging and changes of memory over the life span, studying this relationship across time would add valuable information to this research area and no study has (as far as we know) investigated this longitudinally. This is remiss since improved performance in dual-tasking could have direct implications in real life settings (as suggested by Telner et al. [48]) and thereby increase the quality of life at older ages.

By using data from The Betula study [49–50] we were able to study the relationship between bilingualism and dual-tasking over a follow-up period of 10 years. Unique to this study is that both bilinguals, who have learned their second language in instructed contexts, and monolinguals shared the same native language (Swedish), which may reduce the risk of variances in performance due to smaller vocabulary in one language for the bilinguals. Further, we used matching on possible confounding variables to make the groups as comparable as possible.

In the present study, data from three different dual-tasks conditions were used; (1) free recall with card sorting during encoding, (2) free recall with card sorting during retrieval, and (3) free recall with card sorting during both encoding and retrieval. Simple free recall without card sorting was used as reference task to compute dual-task costs. Based on the assumptions that bilinguals practice switching and monitoring more than monolinguals (e.g., [17–18]) and that these abilities are demanded in dual–tasking situations (e.g., [37, 46]), we expected to find a bilingual advantage in all three dual-task conditions. Since bilinguals that share the same dominant language as monolinguals possess often perform similarly in tasks that require little attentional control [25], we expected groups to be equivalent in the free recall task that was used as reference task.

## Method

### Study population

For this study, data collected within the Betula prospective cohort study [49–50] were used. The Betula project is a study on aging, cognition, and health that started in Umeå, Sweden, in 1988. The participants were selected using stratified randomized sampling (age, sex). To this date, data have been collected over six test waves: 1988–1990 (T1), 1993–1995 (T2), 1998–2000 (T3), 2003–2005 (T4), 2008–2010 (T5), and 2014–2014 (T6). The participants visited the test locations over two sessions at each test wave, mainly giving information about health (Session 1) and cognitive ability (Session 2). So far, six samples have been included in the Betula study; S1 (T1-T6), S2 (T2-T3), S3 (T2-T6), S4 (T3), S5 (T4), and S6 (T5). Age ranges and sample sizes at inclusion have differed between samples. Comparisons between participants in the Betula study and non-participants have shown sufficient population validity with regard to demographic factors such as income, education, and marital status [49]. For the present study, information collected between T2-T4 were used (Sample 1 and 3). Between T4 and T5, unfortunately, there is a large amount of missing data for the cognitive tasks used in this study, and thus, we were not able to include more test waves in the analyses using matched groups while maintaining a decent number of participants in the analyses. The Betula study was approved by the regional Medical Ethical Committee at Umeå University.

### Matching procedure

As a part of a “Language History Questionnaire” participants were requested to indicate (yes/no) to whether they spoke or had studied any other language but Swedish. If the participants indicated knowledge of a second language they then rated, on a scale ranging from very poor (1) to excellent (6), their ability to speak, write, read, and listen to a second language. Those that rated a score of 4 or more on all abilities were considered bilinguals, a procedure previously used (see [26]). Participants that reported knowledge of only one language (i.e. Swedish) and had not studied any other language but Swedish were categorized as monolinguals. A matching procedure was then used to enhance comparison of the groups. Special consideration has to be given to the covariate of years of education wherein the groups are highly unbalanced.

We combined exact matching according to years of education, followed by propensity score matching within those groups, to minimize the effects of potential confounding variables and selection bias, and to make the groups as similar as possible [51]. The propensity score [52], the probability of being a bilingual (vs. monolingual) being conditional on observed covariates, was estimated using logistic regression. A caliper of 0.05 for the propensity score was used in order to achieve balance. Covariates included in the propensity score model were age, sex, study sample (an indicator variable to account for potential effects related to testing experience prior to the first assessment; one vs. two test occasions), and fluid intelligence (G*f*). The WAIS-R Block Design Test [53] was used as indicator of fluid intelligence (G*f*). This is a visuospatial task that requires a set of four, or nine, bicolored blocks. The blocks must be arranged so that they correspond to a target pattern presented to participants by the experimenter. Patterns are presented with increased difficulty, and each pattern has a time limit of either one or two minutes. The maximum number of patterns to solve was ten. The raw score, based on the number of solved patterns and time for this, was used in the matching procedure. A Cronbach’s α of .82 has been reported for Block Design [54].

Matches were obtained for each bilingual, generating a total of 24 matched pairs of participants. No match could be found for 111 bilinguals; these participants was excluded from further analyses.

### Participants

The covariance balance between groups after matching is provided in Table 1. After matching, there were no significant differences between groups in terms of education, age, sex, fluid intelligence, or sample size.

**Table 1:** Covariance balance between language groups after matching.

	Bilinguals	Monolinguals	
	(*n* = 24)	(*n* = 24)	*p-value*
**Age, M (*SD*)**	49.2 (7.75)	50.2 (8.66)	0.48
**Male (%)**	46	46	1
**Years of Education, M *(SD*)**	13.6 (2.61)	13.6 (2.61)	1
**Block Design (*Gf*), M (*SD*)**	30.7 (10.19)	34.2 (9.53)	0.153
**Sample 1 (%)**	50	33	0.153

*Note*: M = mean, SD = Standard Deviation, *Gf* = General fluid ability.

The mean with regard to years of education for the groups was 13.6 (*SD* = 2.61). For age the mean was 49.2 (*SD* = 7.75) and 50.2 (*SD* = 8.66), and for block design (G*f*) 30.7 (*SD* = 10.19) and 34.2 (*SD* = 9.53) for bilinguals and monolinguals, respectively. Among the participants 50% of the bilinguals, and 33% of the monolinguals, belonged to sample 1. Both samples consisted of 46% males.

All bilinguals had English as second language. About 92% of all participants used their second language up to 2 hours a day to listen, read, write, and/or speak. Most bilinguals (94.1%) started to learn their second language within the formal education system, at the age of 9, and studied it at a mean of 6.2 (*SD* = 2.47) years. Thus, most participants categorized as bilinguals in this study are sequential bilinguals since they learned their second language in instructed contexts when starting school.

### Measures

#### Free recall (reference task)

In this immediate memory task, participants were instructed to memorize as many words as possible from a list of 12 nouns. The items were read aloud by the experimenter at a pace of 2 seconds per word. Directly after, participants recalled as many words as possible in any order, but not faster than at a pace of 2 seconds per word. The time interval of 2 seconds was provided by a metronome. Time for recall was 45 seconds and the maximum score was 12. The score was used as single-task score when calculating dual-task costs. Immediate free recall shares many similarities with immediate serial recall [55] and is likely underpinned by a subvocal rehearsal process that is traditionally thought to be carried out by phonological loop mechanism [56]. However, given that the to-be-remembered stimuli were nouns, some semantic processing of the to-be-remembered material is also probable [57].

#### Free recall with card sorting at encoding

The basic memorization procedure was the same as under free recall. But under this condition the participants were told to sort cards while the experimenter read the words. A pack of cards, placed upside down in front of the participants, had either a red or a black square on their front side. Participants turned and placed the cards in two separate packs, one for black squares, and one for red. The cards were sorted at a pace of two seconds, i.e. simultaneously with the encoding of words. When all words had been read, the participants immediately stopped sorting cards and started the retrieval. The number of recalled words and the number of incorrectly sorted cards were counted. The dual-task cost was counted according to the customary formula 100 * (single-task score—dual-task score)/single-task score [58]. Thus, the scores could have both positive and negative values on an individual level.

#### Free recall with card sorting at retrieval

In this condition, participants sorted cards immediately after the experimenter had finished reading the list of words. Cards were sorted over the whole retrieval period (45 sec.), at a pace of two seconds, even when participants could not recall more words.

#### Free recall with card sorting at both encoding and retrieval

Participants were sorting cards during both the encoding and retrieval of words, following the procedure described above.

Word lists (8 versions), and condition orders, were counterbalanced across participants. The words within each list were not duplicated across lists.

### Statistical analysis

We used linear mixed models [59] to analyze the association between bilingualism and 10-year change in cognitive performance. Fixed-effect terms included an overall intercept term, a binary indicator variable for Bilinguals, plus interactions with time. Time was considered as a continuous covariate, measured in decades from baseline (such that the parameter estimate corresponds to a 10-year change). Further, the model included a subject-specific random intercept to account for within-subject correlations between repeated measurements. Statistical analyses were performed in R, version 3.2.2 [60] using the “matching” package [61] to perform the matching, and “lme4” package for the longitudinal analyses. P-values were calculated using Satterthwaite’s approximation [62], which is implemented in the ‘lmerTest’ package.

## Results

**Fig 1:**
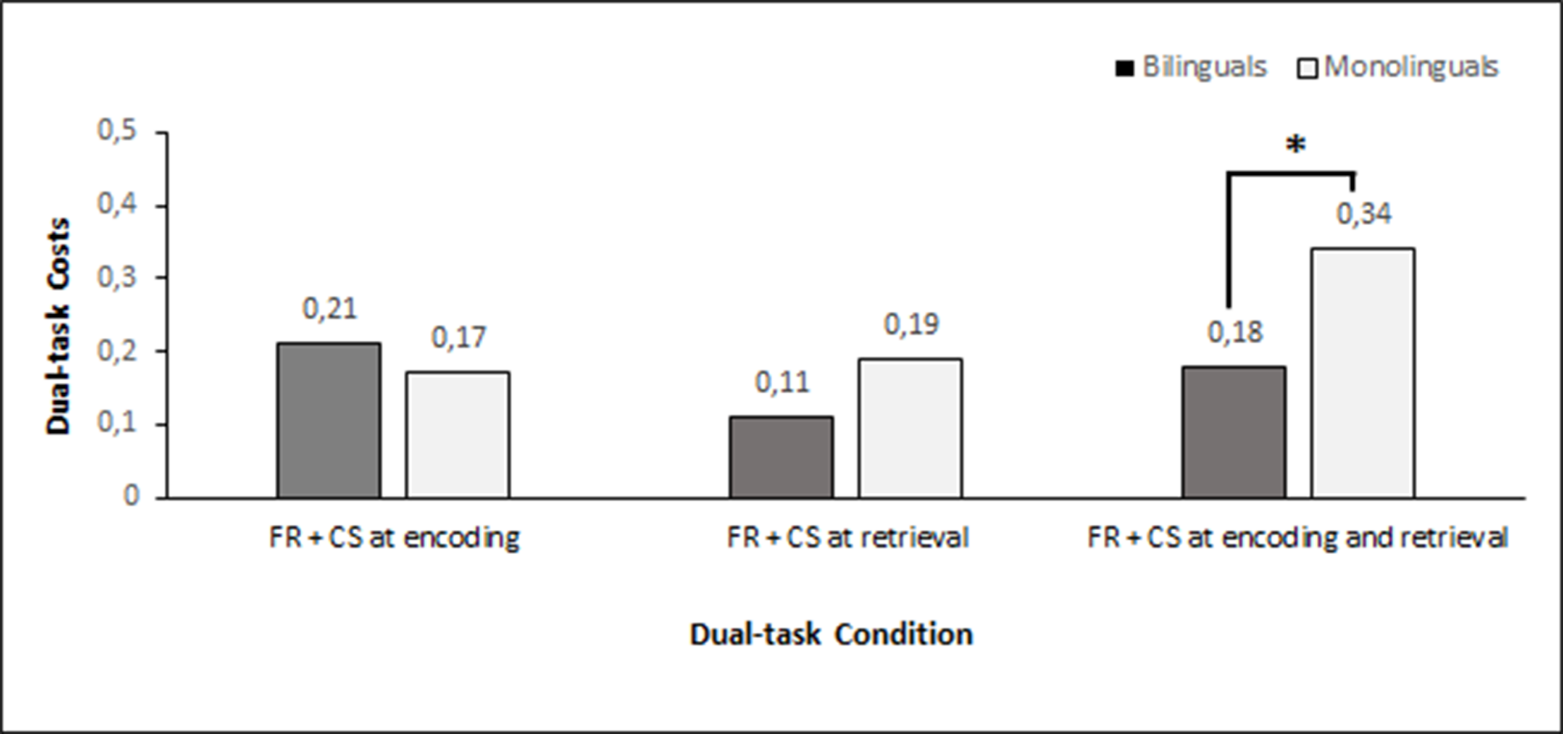
Dual-task costs for both language groups at baseline measurement. Dual-task costs in percentage change from the simple free recall condition. A higher value equals higher dual-task costs. FR = Free Recall; CS = Card Sorting. *p < .05.

The sample included 24 monolinguals and 24 bilinguals that were matched on several variables including age, years of education, sex, fluid intelligence (G*f*), and study sample. The baseline results from the cognitive tasks, as a function of language group, are provided in Fig 1. Results from a student’s t-test showed that bilinguals, at baseline, had less dual-task costs in free recall with card sorting during both encoding and retrieval; *t*(46) = -2.14, *p* = .037. The mean cost for monolinguals was 0.34 (.25), and for bilinguals 0.18 (.24). No difference was found in recall with card sorting during encoding or free recall with card sorting during retrieval only. The number of incorrectly sorted cards was extremely low over all dual-task conditions and test occasions, ranging between mean of 0.00–0.38 incorrectly sorted cards. As expected, there were no differences in the simple free recall task (reference task/no distraction) measuring immediate memory.

**Fig 2:**
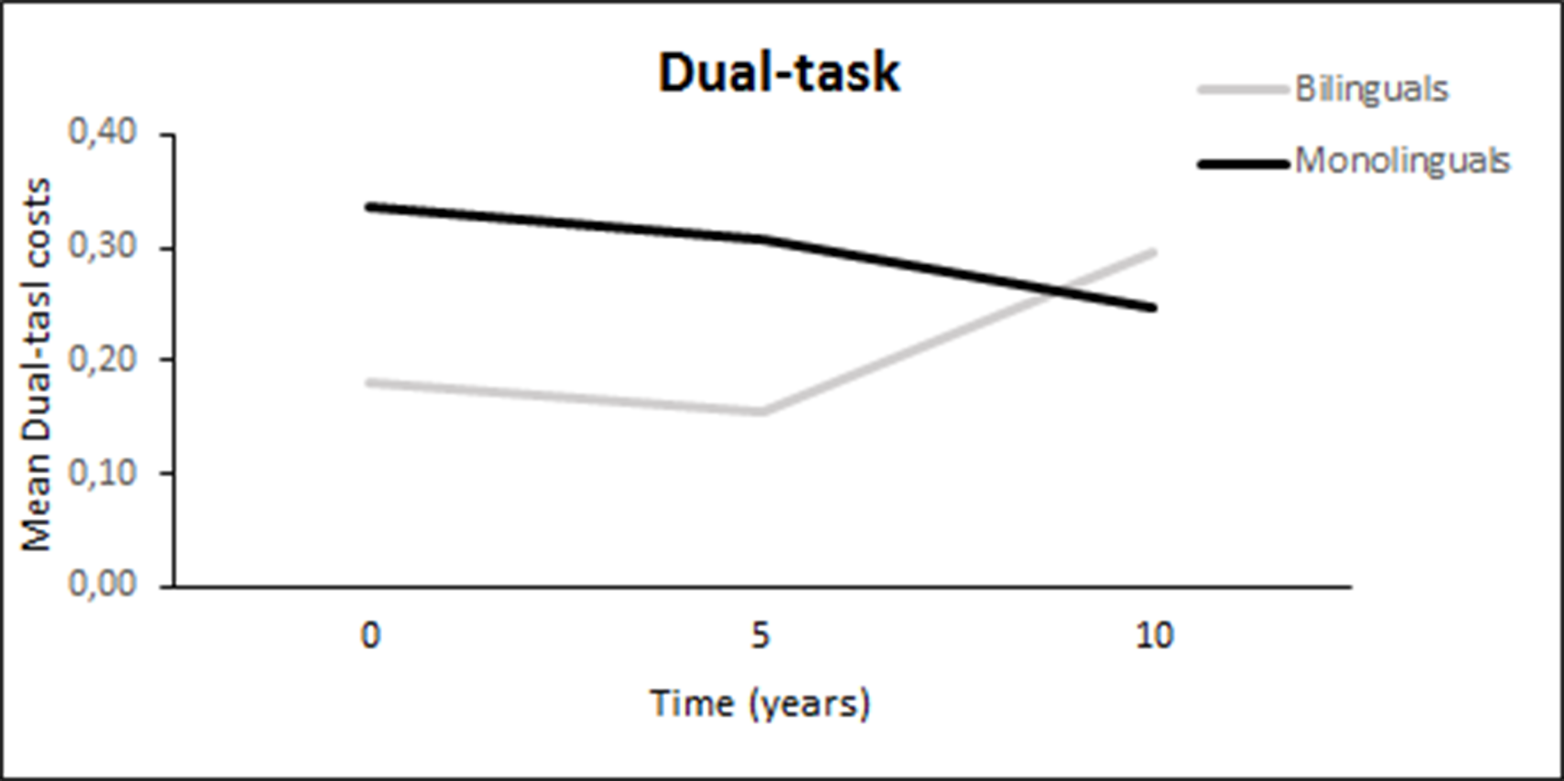
Results from linear mixed models on the effects of bilingualism on dual tasking among participants that were 40–65 years at baseline. Dual task costs in card-sorting during both encoding and retrieval of the word-list. A higher value equals higher dual-task costs.

We modeled the repeated measures of dual-tasks costs in all conditions using linear mixed models. The intercept showed the expected dual tasks costs for a reference person among monolinguals (*b* = 0.34, *SE* = 0.05, *p* < 0.001) in free recall with card sorting during both encoding and retrieval, and a significantly lower cost for bilinguals at baseline (*b* = -0.19, *SE* = 0.07, *p* = 0.006). There was, however, evidence of accelerating age-based dual-task costs (linear trend *b* = 0.20, *SE* = 0.10, *p* = 0.042) among bilinguals over the 10-year period, suggesting that the differences between groups found at baseline do not hold longitudinally (see Fig 2) Results from student’s t-test confirmed that monolinguals (*M* = 0.25, *SD* = 0.22) and bilinguals (*M* = 0.29, *SD* = 0.30) did not significantly differ in performance at the third measurement occasion, 10 years after baseline; t(46) = 0.67, *p* = .51. Significant differences between groups with regard to 10-year change were not found in the other dual-task conditions.

Additional analyses, excluding participants aged 65 years (*n* = 15 in each group), revealed similar results with regard to intercept (*b* = -0.17, *SE* = 0.08, *p* = 0.042), but differences were no longer present for 10-year longitudinal change. Thus, previous findings regarding change may have been driven by the oldest age-group, and speaks against regression to the mean as an explanation for findings. Also in these analyses, no differences were found in any of the other dual task conditions.

## Discussion

Previous research has demonstrated that bilinguals outperform monolinguals in dual-task settings [37, 48]. However, these results are based on cross-sectional data, a common feature in bilingual research (see [63]). Therefore, the aim of this current study was to investigate if there is a bilingual advantage in dual-tasking compared to monolinguals and if it persists over time. Data emanated from a study sample (The Betula study, [49–50]). The bilingual and monolingual sample, 40–65 years at baseline, were matched on several critical factors (age, sex, education, fluid intelligence) that could potentially influence performance in dual-tasking. This study included 10-year follow-up data in three dual-task task conditions. Results from linear mixed models, a statistical method useful in settings where repeated measurements are made on the same statistical units, revealed a significant relationship between bilingualism and dual-tasking in one condition; free recall with card sorting during both encoding and retrieval of words. However, this bilingual advantage was not constant and an interaction was found between bilingualism and change over time, indicating an increase in dual-costs for the bilingual group compared to monolinguals over the 10-year follow-up period. Although descriptively it may look that there even was a bilingual disadvantage at the 10-year follow-up, there were no significant difference between groups at the third time point.

We hypothesized a bilingual advantage in all three dual-task settings. However, this outcome was only present in the most demanding condition (dual-tasking at both encoding and retrieval) and only at baseline. It has previously been shown that the greatest disruptions in memory performance from dual-tasking often occurs during encoding (e.g., [47, 64–66]). Moreover, using a procedure almost identical to that used in the current study, Baddeley et al. [64] found no impact of concurrent card sorting on the free recall of 12 unrelated nouns when the card sorting was performed during recall, while card-sorting during encoding had a reliable disruptive effect. Results from this study, clearly show that neither card-sorting during encoding only, or during retrieval only, was enough to demonstrate differences between bilinguals and monolinguals. Thus, whether the encoding phase or the retrieval phase is most affected by language ability cannot be established from this study. Rather, it is possible that it a cumulative effect of card sorting during both encoding and retrieval was required to demonstrate a bilingual advantage in the sample reported here.

Several studies have shown that age-related changes in executive functioning become more apparent with greater age (e.g., [30, 32–33]). We did not find, however, that the better initial level of performance for bilinguals is beneficial at staving off the apparent decline in cognitive capacities such as executive functioning since bilinguals had a greater decline over 10 years than monolinguals. In the present study, however, some of the participants included in the analyses were close to, or had even passed the age of, 65 at the end of the study. It should be noted that 65 years is the formal retirement age in Sweden. Retirement may, hypothetically, result in less use of the second language, especially in a sample wherein most participants are native Swedish and have learned their second language through education. Many of the bilinguals in this study have used their second language during work and travel which are situations less common after individuals end their professional work. Ljungberg et al. [8] for instance, found no relationship between bilingualism and dementia in their study including individuals from the Betula sample. Thus, less use of the second language after retirement was suggested as a factor that reduced the protective effects of bilingualism. In the same vein, previous research has shown that cognitive stimulation in old age is a factor that may decrease the risk of dementia (e.g., [67–68]). The additional analyses performed, excluding participants aged 65 years at baseline, to some extent confirm this line of reasoning. Despite the small *n* (15) in each group, the results still revealed bilingual advantages at baseline, but no differences were found with regard to change (*p* = .388). However, more longitudinal studies are warranted to investigate whether changes in the use of second language has consequences on cognitive functioning.

This investigation of the longitudinal relationship between bilingualism and dual-tasking is to our knowledge the first of its kind, and provides new and important knowledge to the research field. The present demonstration of a bilingual advantage, although not in the in the long-term, builds on previous cross-sectional results that have found that bilinguals outperform monolinguals in dual-task settings (e.g., [37, 48]. A great strength of this present study was that we matched participants on a number of potential covariates (age, gender, years of education, and fluid intelligence (G*f*)). The use of education as matching variable was deemed necessary in a sample in which many participants have learned their second language within the formal education system. In addition, we matched participants on performance in a visuospatial task (Block design) as indicator of G*f*, a task that is related to executive functioning and frontal lobe activation [69–70], and yet still found bilingual benefits. Previous research has suggested that differences in cognitive functioning, for instance in executive processing, are linked to cultural differences [71]. We minimized the influence of cultural factors within the present study since our sample of bilinguals and monolinguals were native Swedish, and thus have similar cultural backgrounds. In addition, the Betula sample has shown to have a good population of validity based on factors such as employment, education, income, gender, marital status, and number of persons in the home environment [49]. These factors, coupled with results from previous dual-tasking studies in which bilinguals are less likely to have been disadvantaged by their dominant language solving the task [37, 48], give support for a bilingual advantage in dual-tasking.

It should also be stressed that none of the tasks included in this study were initially designed to investigate differences between bilinguals and monolinguals, which also gives further strength to the results. Paap et al. [63] advocate that “a compelling demonstration of a bilingual advantage should show significant advantages on the same component of EF (e.g., monitoring) in two different tasks thus demonstrating convergent validity”. While in the current study, we report the results from a single task—dual-tasking—thought to reflect the executive process of switching [46] convergent validity is provided by similar findings in the context of letter/phonemic fluency whereby the participant is required to produce as many examples of words beginning with a given letter, excluding proper nouns or variants of the same word, within a time limit (e.g., 1 minute). Letter/phonemic fluency requires the executive process of switching between subcategories (e.g., when exemplars within one cluster have been exhausted and the participant is required to shift to a new cluster; [72]). Consistent with the apparent bilingual advantage in the executive process of switching reported here and again using the Betula longitudinal sample, Ljungberg et al. [26] report a bilingual advantage at the first testing session and across time. Importantly, no bilingual advantage was found in a category fluency task (producing names of occupations) that made similar demands to the letter fluency task in terms of other executively demanding processes (e.g., self-monitoring and inhibition of previously produced responses, inhibition of irrelevant responses, and organization of verbal retrieval) but has less requirement in terms of switching [72] Therefore, coupled with the study of Ljungberg et al [26], the current study supports the notion of a bilingual advantage in relation to the executive process of switching, at least from a cross-sectional perspective.

An interesting aspect of this study is that the dependent variable in all conditions, number of recalled words, was based on lexical information. Previous studies have shown that bilinguals are disadvantaged in tasks that place greater demands on lexical access (e.g., [47, 73]). For instance, Fernandes et al. [47] found that bilinguals performed worse in a simple free recall task (no distraction), and under conditions of distraction at encoding and retrieval of words. However, all participants in our sample had Swedish as first language, a factor that, potentially, reduced negative effects of smaller vocabulary size among bilinguals. It has been shown that monolinguals and bilinguals that share the same native language and have similar vocabulary knowledge in their first language perform similarly in phonological short-term memory tasks [15]. Similarly, and as expected from our hypothesis, we found no evidence that bilingualism served to increase performance in a simple free recall task. Thus, our data support the notion that previous differences found between bilinguals and monolinguals in immediate memory are influenced by vocabulary size in the language used to measure immediate memory.

It should be stressed that our distraction task (card-sorting) probably became a relatively automated process for most participants. This assumption is, to some extent, supported by the fact that participants overall made very few errors in card sorting. Still, although we do not know how cognitively demanding participants experienced dual-tasking, cognitive load certainly increased since overall memory performance decreased when card-sorting was added. To mix a relatively automated task with one of verbal character as in the current study, is similar to the study of Telner et al. [48]. They investigated the number of driver mistakes while performing a number of verbal tasks. In their study, the focus was on measuring driving performance, which may be regarded as a relatively automated process. In our study, the focus was mainly on verbal performance. However, the conclusions, at least in short-term, are rather consistent, i.e. bilinguals often perform better that monolinguals when they execute a verbal task and relatively automated process simultaneously, possibly because these tasks are conceptually independent of each other.

Despite several strengths of this present study, some minor limitations should be acknowledged. Most of our bilingual participants (92%) used their second language 0–2 hours per day, which is a broad time interval. For example, there is large difference between using a second language for a few minutes a day compared to almost two hours a day. However, even if many of the bilingual participants in our sample did not use their second language frequently, it seems that the subjective estimation of language proficiency was enough to generate different outcomes in cognitive performance. Further, even if the monolingual participants confirmed that they could not speak or had studied any other language but Swedish, we cannot rule out the possibility that they occasionally had come into contact with another language than Swedish (e.g. by travel, TV-watching, internet). But gained knowledge of single words and/or phrases learned through such experiences is markedly different from fluent knowledge in a second language, and thus, it is most likely that language use was not a factor that influenced performance in the monolingual group. Based on this, and also considering the age groups included in the present study, the risk seems small that practice of a second language was a factor that influenced dual-tasking performance during the follow-up period. In the bilingual group, however, and as discussed earlier, there is a potential possibility that the deterioration in dual-tasking performance present at the 10-year follow-up may reflect less use of the second language as a consequence of retirement age. However, the use of a more sensitive instrument may have altered the results. Recently, Luk and Bialystok [74] suggested that bilingualism should not to be considered as a categorical variable, it should rather be regarded from a multidimensional perspective, with greater understanding for individual variation in aspects of bilingual experience.

It should also be noted that our results are restricted to bilinguals where most have learned their second language through the formal education system. It is perhaps problematic to compare such bilinguals, sometimes referred to as sequential bilinguals, with native bilinguals, or simultaneous bilinguals, that have practiced two languages from birth and often have a more balanced proficiency between their primary and a second language. If this is so, then the results from this study may not be generalized across all bilinguals groups. However, since it is well documented that being bilingual from birth is advantageous for the development of the executive control system [10], and it recently has been found that also sequential bilingual children outperform monolingual children in attentional control task [75], it is highly plausible that the results from this study can be transferred across bilingual groups, and that native bilinguals would have performed at an even higher level compared to the groups included in this study.

In conclusion, the results from this study demonstrate that bilinguals can outperform monolinguals in dual-task settings. Differences found at baseline seem to go beyond the influence of age, sex, education and fluid intelligence (G*f*) in a sample where most participants had learned their second language through education. However, these differences were not stable over a period of 10 years and do not support the notion that bilingualism can enhance cognitive performance that in turn may postpone the onset of cognitive impairment when entering old age. However, for this study, performance in dual-tasking was still restricted to an age group that had not yet entered higher ages at the end of the study, and thus, future possible benefits for the bilingual sample are unclear. Finally, although the outcome of the present study indicated a bilingual advantage cross-sectionally, that bilinguals had a greater decline over time may depend on the infrequent use of second language when reaching retirement age.

## References

[1] Bhatia TK, & Ritchie WC. The handbook of bilingualism and multilingualism (2nd ed.). 2013 Oxford: Wiley-Blackwell doi: 10.1002/9781118332382

[2] Bialystok E, Craik FIM, Freedman M. Bilingualism as a protection against the onset of symptoms of dementia. Neuropsychologia. 2007;45(2):459–64. doi: 10.1016/j.neuropsychologia.2006.10.009 17125807 10.1016/j.neuropsychologia.2006.10.009

[3] Bialystok E, Craik FIM, Freedman M. Delaying the onset of Alzheimer disease. Neurology. 2010;75:1726–9. doi: 10.1212/WNL.0b013e3181fc2a1c 21060095 10.1212/WNL.0b013e3181fc2a1c PMC3033609

[4] Ossher L, Bialystok E, Craik FIM, Murphy KJ, Troyer AK. The effect of bilingualism on amnestic mild cognitive impairment. The journals of gerontology. Series B: Psychological sciences and social sciences. 2013;68(1):8–12. doi: 10.1093/geronb/gbs038 22454387 10.1093/geronb/gbs038 PMC3991134

[5] Chertkow H, Whitehead V, Phillips N, Wolfson C, Atherton J, Bergman H. Multilingualism (but not always bilingualism) delays the onset of Alzheimer disease: Evidence from a bilingual community. Alzheimer's Disease & Associated Disorders. 2010;24(2):118–25. doi: 10.1097/WAD.0b013e3181ca1221 20505429 10.1097/WAD.0b013e3181ca1221

[6] Lawton DM, Gasquoine PG, Weimer AA. Age of dementia diagnosis in community dwelling bilingual and monolingual Hispanic Americans. Cortex. 2014;66:141–5. doi: 10.1016/j.cortex.2014.11.017 25598395 10.1016/j.cortex.2014.11.017 PMC4426973

[7] Yeung CM, St John PD, Menec V, Tyas SL. Is bilingualism associated with a lower risk of dementia in community-living older adults? Cross-sectional and prospective analyses. Alzheimer Disease & Associated Disorders. 2014;28(4):326–32. doi: 10.1097/WAD.0000000000000019 24614266 10.1097/WAD.0000000000000019

[8] Ljungberg JK, Hansson P, Adolfsson R, Nilsson L-G. The effect of language skills on dementia in a Swedish longitudinal cohort. Linguistic Approaches to Bilingualism. 2016;6(1/2):190–204. doi: 10.1075/lab.14031.lju

[9] Adesope OO, Lavin T, Thompson T, Ungerleider C. A Systematic Review and Meta-Analysis of the Cognitive Correlates of Bilingualism. Review of Educational Research. 2010;80(2):207–45. doi: 10.3102/0034654310368803

[10] Bialystok E, Craik F, Green D, Gollan T. Bilingual Minds. Psychological Science in the Public Interest. 2009;10(3):89–129. doi: 10.1177/1529100610387084 26168404 10.1177/1529100610387084

[11] Hilchey MD, Klein RM. Are there bilingual advantages on nonlinguistic interference tasks? Implications for the plasticity of executive control processes. Psychonomic Bulletin & Review. 2011;18(4):625–58. doi: 10.3758/s13423-011-0116-7 21674283 10.3758/s13423-011-0116-7

[12] Bialystok E, Poarch G, Luo L, Craik FIM. Effects of Bilingualism and Aging on Executive Function and Working Memory. Psychology and Aging. 2014;29(3):696–705. doi: 10.1037/a0037254 25244487 10.1037/a0037254 PMC4274603

[13] Hernández M, Costa A, Fuentes LJ, Vivas AB, Sebastián-Gallés N. (2010). The impact of bilingualism on the executive control and orienting networks of attention. Bilingualism: Language and Cognition, 2010;13, 315–325. doi: 10.1017/S1366728909990010

[14] Costa A, Hernández M, Costa-Faidella J, Sebastián-Gallés N. On the bilingual advantage in conflict processing: Now you see it, now you don’t. Cognition. 2009;113(2):135–49. doi: 10.1016/j.cognition.2009.08.001 19729156 10.1016/j.cognition.2009.08.001

[15] Pelham SD, Abrams L. Cognitive advantages and disadvantages in early and late bilinguals. Journal of Experimental Psychology: Learning, Memory, and Cognition. 2014;40(2):313–25. doi: 10.1037/a0035224 24294916 10.1037/a0035224

[16] Bialystok E, Craik FIM, Klein R, Viswanathan M. Bilingualism, aging, and cognitive control: Evidence from the Simon task Psychology and Aging. 2004;19(2):290–303. doi: 10.1037/0882-7974.19.2.290 15222822 10.1037/0882-7974.19.2.290

[17] Prior A, Gollan TH. Good Language-Switchers are Good Task-Switchers: Evidence from Spanish–English and Mandarin–English Bilinguals. Journal of the International Neuropsychological Society. 2011;17(4):682–91. doi: 10.1017/S1355617711000580 22882810 10.1017/S1355617711000580

[18] Wiseheart M, Viswanathan M, Bialystok E. Flexibility in task switching by monolinguals and bilinguals. Bilingualism: Language and Cognition. 2016;19(1):141–6. doi: 10.1017/S1366728914000273 26877705 10.1017/S1366728914000273 PMC4749032

[19] de Bruin A, Treccani B, Della Sala S. Cognitive advantage in bilingualism: an example of publication bias? Psychological Science. 2015;26(1):99–107. doi: 10.1177/0956797614557866 25475825 10.1177/0956797614557866

[20] Donnelly S, Brooks PJ, Homer BD. (2015). Examining the Bilingual Advantage on Conflict Resolution Tasks: A Meta-Analysis. Proceedings of the 37th Annual Conference of the cognitive Science Society. 2015. Austin, TX: Cognitive Science Society.

[21] Paap KR, Johnson HA, Sawi O. Bilingual advantages in executive functioning either do not exist or are restricted to very specific and undetermined circumstances. Cortex. 2015;69:265–78. doi: 10.1016/j.cortex.2015.04.014 26048659 10.1016/j.cortex.2015.04.014

[22] von Bastian CC, Souza AS, Gade M. No evidence for bilingual cognitive advantages: A test of four hypotheses. Journal of Experimental Psychology. 2015;145(6):144 doi: 10.1037/xge0000120 26523426 10.1037/xge0000120

[23] Bialystok E, Luk G. Receptive vocabulary differences in monolingual and bilingual adults. Bilingualism: Language and Cognition. 2012;15(2):397–401. doi: 10.1017/S136672891100040X 10.1017/S1366728909990423 PMC4349351 25750580

[24] Bialystok E, Luk G, Peets KF, Yang S. Receptive vocabulary differences in monolingual and bilingual children. Bilingualism: Language and Cognition. 2010;13(4):525–31. doi: 10.1017/S1366728909990423 25750580 10.1017/S1366728909990423 PMC4349351

[25] Kaushanskaya M, Blumenfeld HK, Marian V. The relationship between vocabulary and short-term memory measures in monolingual and bilingual speakers. International Journal of Bilingualism. 2011;15(4):408–25. doi: 10.1177/1367006911403201 22518091 10.1177/1367006911403201 PMC3328198

[26] Ljungberg JK, Hansson P, Andrés P, Josefsson M, Nilsson LG. A Longitudinal Study of Memory Advantages in Bilinguals. PLoS One. 2013;8(9): e73029 doi: 10.1371/journal.pone.0073029 24023803 10.1371/journal.pone.0073029 PMC3762844

[27] Rönnlund M, Nyberg L, Bäckman L, Nilsson L-G. Stability, growth, and decline in adult life span development of declarative memory: Cross-sectional and longitudinal data from a population-based study. Psychology and Aging. 2005;20(1):3–18. doi: 10.1037/0882-7974.20.1.3 15769210 10.1037/0882-7974.20.1.3

[28] Park DC, Payer D. Working memory across the adult lifespan In Bialystok E. & Craik F.I.M. (Eds.), Lifespan Cognition: Mechanisms of Change. 2006 (pp. 128–142). New York, NY: Oxford University Press.

[29] Sander MC, Lindenberger U, Werkle-Bergner M. Lifespan age differences in working memory: A two-component framework. Neuroscience & Biobehavioral Reviews. 2012;36(9):2007–33. doi: 10.1016/j.neubiorev.2012.06.004 22771333 10.1016/j.neubiorev.2012.06.004

[30] Zelazo PD, Craik FIM., Booth L. Executive function across the life span. Acta Psychologica. 2004;115(2–3):167–83. doi: 10.1016/j.actpsy.2003.12.005 14962399 10.1016/j.actpsy.2003.12.005

[31] Mayr U, Liebscher T. Is there an age deficit in the selection of mental sets? European Journal of Cognitive Psychology. 2001;13(1–2):47–69. doi: 10.1080/09541440042000214

[32] Salthouse TA, Miles JD. Aging and time-sharing aspects of executive control. Memory & Cognition. 2002;30(4):572–82. doi: 10.3758/BF03194958 12184558 10.3758/bf03194958

[33] Treitz FH, Heyder K, Daum I. Differential Course of Executive Control Changes During Normal Aging. Aging, Neuropsychology, and Cognition. 2007;14(4):370–93. doi: 10.1080/13825580600678442 17612814 10.1080/13825580600678442

[34] Connelly SL, Hasher L, Zacks RT. Age and reading: The impact of distraction. Psychology and Aging. 1991;6(4):533–41. 1777141 10.1037//0882-7974.6.4.533

[35] Abutalebi J, Green DW. Control mechanisms in bilingual language production: Neural evidence from language switching studies. Language and Cognitive Processes. 2008;23(4):557–82. doi: 10.1080/01690960801920602

[36] Green DWD. Mental control of the bilingual lexico-semantic system. Bilingualism: Language and Cognition. 1998;1(2):67–81. doi: 10.1017/S1366728998000133

[37] Bialystok E, Craik FIM, Ruocco AC. Dual-modality monitoring in a classification task: the effects of bilingualism and ageing. Quarterly Journal of Experimental Psychology. 2006;59(11):1968–83. doi: 10.1080/17470210500482955 16987784 10.1080/17470210500482955

[38] Poarch GJ, Bialystock E. Bilingualism as a model for multitasking. Developmental Review. 2015;35:113–24. doi: 10.1016/j.dr.2014.12.003 25821336 10.1016/j.dr.2014.12.003 PMC4371212

[39] Abutalebi J, Della Rosa PA, Green DW, Hernandez M, Scifo P, Keim R, et al Cerebral Cortex cingulate cortex for conflict monitoring. Cerebral Cortex. 2012;22(9):2076–86. 22038906 10.1093/cercor/bhr287

[40] Beste C, Ness V, Lukas C, Hoffmann R, Stüwe S, Falkenstein M, et al Mechanisms mediating parallel action monitoring in fronto-striatal circuits. Neuroimage. 2012;62(1):137–46. doi: 10.1016/j.neuroimage.2012.05.019 22617828 10.1016/j.neuroimage.2012.05.019

[41] Cattaneo G, Calabria M, Marne P, Gironell A, Abutalebi J, Costa A. The role of executive control in bilingual language production: A study with Parkinson’s disease individuals. Neuropsychologia. 2015;66:99–110. doi: 10.1016/j.neuropsychologia.2014.11.006 25448860 10.1016/j.neuropsychologia.2014.11.006

[42] Abutalebi J, Annoni JM, Zimine I, Pegna AJ, Seghier ML, Lee-Jahnke H, et al Language control and lexical competition in bilinguals: An event-related fMRI study. Cerebral Cortex. 2008;18(7):1496–505. doi: 10.1093/cercor/bhm182 17947346 10.1093/cercor/bhm182

[43] Verhaeghen P, Cerella J. Aging, executive control, and attention: a review of meta-analyses. Neuroscience and Biobehavioral Reviews. 2002;26(7):849–57. doi: 10.1016/S0149-7634(02)00071-4 12470697 10.1016/s0149-7634(02)00071-4

[44] McIsaac TL, Lamberg EM, Muratori LM. Building a framework for a dual task taxonomy. BioMed Research International. 2015;pl. doi: 10.1155/2015/591475 25961027 10.1155/2015/591475 PMC4417581

[45] Bialystok E. Reshaping the mind: The benefits of bilingualism. Canadian Journal of Experimental Psychology. 2011;65(4):229–35. doi: 10.1037/a0025406 21910523 10.1037/a0025406 PMC4341987

[46] Salvucci DD, Taatgen NA, Borst J. Toward a unified theory of the multitasking continuum: From concurrent performance to task switching, interruption, and resumption. In Human factors in computing systems: CHI 2009 conference proceedings (pp. 1819–1828).

[47] Fernandes MA, Craik F, Bialystok E, Kreuger S. Effects of bilingualism, aging, and semantic relatedness on memory under divided attention. Canadian Journal of Experimental Psychology. 2007;61(2):128–41. doi: 10.1037/cjep2007014 17665753 10.1037/cjep2007014

[48] Telner JA, Wiesenthal DL, Bialystok E, York M. Is There a Bilingual Advantage When Driving and Speaking Over a Cellular Telephone? Proceedings of the Human Factors and Ergonomics Society 52nd Annual Meeting. 2008;52(23):1905–9. doi: 10.1177/15419312080520231

[49] Nilsson L, Adolfsson R, Bäckman L, Frias CM, Molander B, Nyberg L. The Betula prospective cohort study: Memory, health, and aging. Aging Neuropsychology and Cognition. 1997;11(2–3):134–48. doi: 10.1080/13825589708256633

[50] Nilsson L-G, Adolfsson R, Bäckman L, de Frias CM, Molander B, Nyberg L. Betula: A prospective cohort study on memory, health and aging. Aging, Aging, Neuropsychology, and Cognition. 2004;11(2–3):134–48. doi: 10.1080/13825580490511026

[51] Stuart EA. Matching methods for causal inference: A review and a look forward. Statistical science: a review journal of the Institute of Mathematical Statistics. 2010;25(1):1–21. doi: 10.1214/09-STS313 20871802 10.1214/09-STS313 PMC2943670

[52] Austin PC. An Introduction to Propensity Score Methods for Reducing the Effects of Confounding in Observational Studies. Multivariate behavioral research. 2011;46(3):399–424. doi: 10.1080/00273171.2011.568786 21818162 10.1080/00273171.2011.568786 PMC3144483

[53] Wechsler D. Wechsler adult intelligence scale-Revised. 1981 San Antonio, SA: Psychological Corporation.

[54] Rönnlund M, Nilsson L-G. The Betula study: Reliabilities and Long-Term Stabilities of Memory Test Performances Over the Adult Lifespanaltic Journal of Psychology. 2006;7:6–14.

[55] Bhatarah P, Ward G, Smith J, Hayes L. Examining the relationship between free recall and immediate serial recall: Similar patterns of rehearsal and similar effects of word length, presentation rate, and articulatory suppression. Memory & Cognition. 2009:37(5): 689–713. doi: 10.3758/MC.37.5.689 19487760 10.3758/MC.37.5.689

[56] Baddeley AD. Working Memory. 1986 Oxford, UK: Oxford University Press.

[57] Howard MW, Kahana MJ. When does semantic similarity help episodic retrieval?. Journal of Memory and Language. 2002:46(1): 85–98. doi: 10.1006/jmla.2001.2798

[58] McDowd J. The effects of age and extended practice on divided attention performance. Journal of Gerontology. 1986;41:764–769. doi: 10.1093/geronj/41.6.764 3772053 10.1093/geronj/41.6.764

[59] Verbeke G, Molenberghs G. Linear mixed models for longitudinal data 2009 New York: Springer Science & Business Media.

[60] R Core Team. R: A language and environment for statistical computing R Foundation for Statistical Computing 2015 Vienna, Austria https://www.R-project.org/

[61] Sekhon JS. Multivariate and Propensity Score Matching Software with Automated Balance Optimization: The Matching package for R. Journal of Statistical Software. 2011;42(7):127–210. doi: 10.18637/jss.v042.i07

[62] Goodnight JH. Tests of hypotheses in fixed effects linear models. Communications in Statistics—Theory and Methods. 1980;9:167–180. doi: 10.1080/03610928008827869

[63] Paap KR, Johnson HA, Sawi O. Are bilingual advantages dependent upon specific tasks or specific bilingual experiences? Journal of Cognitive Psychology. 2014;26(6):615–39. doi: 10.1080/20445911.2014.944914

[64] Baddeley A, Lewis V, Eldridge M, Thomson N. Attention and retrieval from long-term memory. Journal of Experimental Psychology: General. 1984;113(4):518–40. 540. doi: 10.1037/0096-3445.113.4.518

[65] Craik FI, Govoni R, Naveh-Benjamin M, Anderson ND. The effects of divided attention on encoding and retrieval processes in human memory. Journal of Experimental Psychology: General. 1996;125(2):159–80. doi: 10.1037/0096-3445.125.2.159 8683192 10.1037//0096-3445.125.2.159

[66] Fernandes MA, Moscovitch M. Divided attention and memory: Evidence of substantial interference effects at retrieval and encoding. Journal of Experimental Psychology: General. 2000;129(2):155–76. 10868332 10.1037//0096-3445.129.2.155

[67] Karp A, Paillard-Borg S, Wang HX, Silverstein M, Winblad B, Fratiglioni L. Mental, physical and social components in leisure activities equally contribute to decrease dementia risk. Dementia and Geriatric Cognitive Disorders. 2006;21(2):65–73. doi: 10.1159/000089919 16319455 10.1159/000089919

[68] Paillard-Borg S, Fratiglioni L, Winblad B, Wang HX. Leisure activities in late life in relation to dementia risk: Principal component analysis. Dementia and Geriatric Cognitive Disorders. 2009;28(2):136–44. doi: 10.1159/000235576 19690416 10.1159/000235576

[69] Lezak MD, Howieson DB, Loring DW. Neuropsychological assessment (4th Edition). 2004 Oxford, UK; Oxford University Press.

[70] Wallesch C-W, Curio N, Galazky I, Jost S, Synowitz H. The Neuropsychology of Blunt Head Injury in the Early Postacute Stage: Effects of Focal Lesions and Diffuse Axonal Injury. Journal of Neurotrauma. 2001;18(1):2001 doi: 10.1089/089771501750055730 11200246 10.1089/089771501750055730

[71] Morton JB. Language, bilingualism, and executive functioning in early development. Psychological Reports. 2010;107(3):888–90. doi: 10.2466/04.11.28.PR0.107.6.888-890 21323147 10.2466/04.11.28.PR0.107.6.888-890

[72] Troyer AK, Moscovitch M, Winocur G. Clustering and switching as two components of verbal fluency: Evidence from younger and older healthy adults. Neuropsychology. 1997;11(1):138–46. doi: 10.1037//0894-4105.11.1.138 9055277 10.1037//0894-4105.11.1.138

[73] Gollan TH, Montoya RI, Werner GA. Semantic and letter fluency in Spanish-English bilinguals. Neuropsychology. 2002;16(4):562–76. doi: 10.1037/0894-4105.16.4.562 12382994

[74] Luk G, Bialystok E. Bilingualism is not a categorical variable: Interaction between language proficiency and usage. Journal of Cognitive Psychology. 2013;25(5):605–21. doi: 10.1080/20445911.2013.795574 24073327 10.1080/20445911.2013.795574 PMC3780436

[75] Kalashnikova M, Mattock K. Maturation of executive functioning skills in early sequential bilingualism. International Journal of Bilingual Education and Bilingualism. 2014;17(1):111–23. doi: 10.1080/13670050.2012.746284

